# Supplementary material for: Oleate Impacts on Acetoclastic and Hydrogenotrophic Methanogenesis under Mesophilic and Thermophilic Conditions
Source: Int J Environ Res Public Health. 2023 Feb 15;20(4):3423. doi: 10.3390/ijerph20043423 (PMC9960261; doi:10.3390/ijerph20043423)
Supplement: Supplementary file 1 [file ijerph-20-03423-s001.zip › ijerph-2200994-supplementary.pdf]

Supplementary Materials

Table S1 Detailed elements in phosphate buffer

Element	Concentration (mg/L)	Element	Concentration (mg/L)
$MgCl_2 \cdot 6H_2O$	400	$CoCl_2 \cdot 6H_2O$	0.17
$CaCl_2 \cdot 2H_2O$	150	$FeCl_2 \cdot 4H_2O$	2
NH_4Cl	500	$NaHCO_3$	5000
K_2HPO_4	872	$ZnCl_2$	0.07
KH_2PO_4	680	H_3BO_3	0.06
$MnCl_2 \cdot 4H_2O$	0.611	$NiCl_2 \cdot 6H_2O$	0.04
$CuCl_2 \cdot 2H_2O$	0.027	$Na_2MoO_4 \cdot 2H_2O$	0.025
EDTA	5		

Table S2 Primary bacteria composition in mesophilic and thermophilic sludge

Temperature	Phylum	Genus	Number of OTU	Percentage (%)	Similarity (%)
Mesophilic	*Thermotogae*	*S1*	6044	14.14	100
	Bacteroidetes	*Bacteroides*	5272	12.33	99.92
		Others	8698	20.35	
	Firmicutes	*Streptococcus*	4424	10.35	97.82
		Syntrophomonas	2210	5.17	97.7
		Others	6512	15.23	
	Actinobacteria	*Actinomyces*	4247	9.93	98.02
	Chloroflexi	*T78*	3733	8.73	100
	Synergistetes	*vadinCA02*	895	2.09	96.15
		HA73	716	1.67	98.47
	Total		42751	100.00	
Thermophilic	*Thermotogae*	*S1*	26573	59.04	100
	Bacteroidetes	*Others*	5591	12.42	
	Firmicutes	*Coprothermobacter*	2667	5.93	100
		Thermacetogenium	1365	3.03	97.96
		Sporanaerobacter	663	1.47	99.47
		Others	5076	11.28	
	Synergistetes	*Anaerobaculum*	2095	4.66	99.82
	OP9	*Others*	975	2.17	99.79
	Total		45005	100.00	
